# Interstitial Pneumonia with Autoimmune Features (IPAF): A Single-Centre, Prospective Study

**DOI:** 10.31138/mjr.31.3.330

**Published:** 2020-09-30

**Authors:** Maria Karampeli, Konstantinos Thomas, Sofia Flouda, Aikaterina Chavatza, Dionisios Nikolopoulos, Antigone Pieta, Dimitrios Tseronis, Michail Aggelakos, Dimitra Kassara, Vasiliki Tzavara, Pelagia Katsimbri, Dimitrios Boumpas, Theofanis Karageorgas

**Affiliations:** 1 Department of Internal Medicine, General Hospital Korgialenio-Benakio HRC, Athens, Greece; 2 4^th^ Department of Internal Medicine, “Attikon” University Hospital of Athens, Greece; 3 Rheumatology and Clinical Immunology Unit, 4^th^ Department of Internal Medicine, “Attikon” University Hospital of Athens, Greece

**Keywords:** Interstitial lung diseases, connective tissue diseases, interstitial pneumonia with autoimmune features, pulmonary fibrosis

## Abstract

**Objectives:**

Interstitial pneumonia with autoimmune features (IPAF) refers to patients with interstitial lung disease and autoimmune features not fulfilling the classification criteria for a specific connective tissue disease. We sought to study the characteristics, disease progression, response to treatment and complications of patients with IPAF in 1-year follow-up period.

**Methods:**

Clinical and laboratory findings, comorbidities, medications, pulmonary function tests (PFTs), chest HRCT and complications during the one-year follow-up period were documented for each of the 39 enrolled patients with IPAF.

**Results:**

The mean age at the time of IPAF diagnosis was 63.2 (±11) years, and 62% of patients were female. The most common clinical features were arthritis (82%) and rash (54%-not included in the IPAF criteria). Antinuclear antibodies (ANA) (59%) and non-specific interstitial pneumonia (NSIP-61.5%) were the most prevalent autoantibodies and radiological pattern respectively. PFTs at 12 months from baseline stabilized or improved in 79.5% of patients (p> 0.05). Infections were observed in 23.1% of patients during the first and in 12.8% during the second semester of follow-up. Two patients (5.1%) required hospitalization. All infections occurred in patients with non-usual interstitial pneumonia (UIP) pattern (p=0.02).

**Conclusions:**

Arthritis and rash are among the most common features in IPAF suggesting rash could be included into IPAF criteria. Almost 80% of patients had stable/improved PFTs at the end of follow-up. Infections occurred mainly in the first semester of treatment and in patients with non-UIP radiological pattern probably due to higher doses of corticosteroids used in these patients.

## INTRODUCTION

Interstitial lung diseases (ILD) form an heterogenous group of disorders with a crude prevalence of approximately 98 cases/100000 people. More than half of patients with ILD secondary to known causes are due to a defined connective tissue disease (CTD).^1,2^ However, a substantial proportion of patients with secondary ILD present clinical and laboratory features suggesting an autoimmune condition, but without meeting the classification criteria for a specific CTD.^3^ Various attempts have been made in the past to study this subgroup of ILD patients using different sets of criteria.^4–7^ In 2015, a joint European Respiratory Society and American Thoracic Society (ERS/ATS) meeting coined the term interstitial pneumonia with autoimmune features (IPAF) and proposed a new set of classification criteria in order to facilitate and promote the research regarding this subgroup of patients. Classification criteria for IPAF fall into three distinct domains, namely, clinical, serological, and morphological.^8^

Most data regarding IPAF derive mainly from retrospective^9–13^ and few prospective studies.^14,15^ However, there are still many aspects of IPAF, concerning epidemiology, clinical features, natural history and prognosis of this condition, that remain ill-defined.^16^

The aim of this prospective follow-up study is to describe, for the first time in Greece, the epidemiological and clinical characteristics of patients with IPAF and to observe disease progression, response to treatment and infection rate in 1-year follow-up period.

## MATERIALS AND METHODS

Our cohort included 39 patients fulfilling the ERS/ATS 2015 IPAF criteria.^8^ These were prospectively followed from November 2015 to September 2019 at the Rheumatology and Clinical Immunology Unit of “Attikon” University Hospital of Athens. The study was performed according to the WMA Declaration of Helsinki, approved by the local Ethics Committee (EBD 103), and all patients provided informed consent.

Patients with defined CTD or other known aetiology for ILD were excluded. ILD diagnosis was established by high resolution computed tomography of the chest (HRCT) and/or surgical lung biopsy (when available). For each patient, the initial dataset included demographical data (age, gender, profession); comorbidities (diabetes mellitus, hypertension, dyslipidaemia, BMI, smoking, COPD/asthma, coronary artery disease, cerebrovascular accident, malignancy, gastro-oesophageal reflux disease, osteoporosis, thyroid disease, tuberculosis, hepatitis B and C); clinical and laboratory data regarding IPAF (disease duration, extra-pulmonary manifestations, ANA, extractable nuclear antigens [ENAs], double-stranded DNA [dsDNA], rheumatoid factor [RF] and anti-citrullinated protein/peptide antibody (ACPA) titers, anti-Jo1, myositis-related autoantibodies); and past and current medications use (of relevance to IPAF). Additional data regarding ILD included date of ILD diagnosis, presenting symptoms as well as the subjective respiratory functional status (as per Medical Research Council Dyspnoea Scale - MRC), HRCT pattern of ILD (classification in UIP, NSIP, OP, LIP, overlap), and the results of PFTs (forced vital capacity [FVC], forced expiratory volume [FEV1], diffusion capacity [DLco], total lung capacity [TLC] reported in % of predicted values and corrected for age, gender and height).

During the follow-up visits (every 3 months during the first year) treatment and dose modifications (csDMARDs, bDMARDs, corticosteroids), adverse events (infections, hospitalizations or other events attributed either to the disease itself or to its treatment) as well as data regarding ILD (patient’s respiratory functional status as per MRC, PFTs and HRCT) were recorded.

Baseline data were analysed by descriptive statistics with categorical variables being expressed as counts and percentages. Normally distributed continuous variables were expressed as mean (standard deviation, SD), whereas non-normally distributed variables were presented as median (interquartile range, IQR). Comparisons of continuous variables with normal distribution were performed using paired *t*-test, while for those with non-normal distribution, a Mann-Whitney test was adopted. Comparisons of categorical variables were performed using chi square (*x*^2^) test. Univariate models were performed in order to identify determinants of infection and clinically significant difference in PFTs (defined as change of ≥ 10% in FVC and/or ≥ 15% in DLCO).^17^ A p-value <0.05 was set as threshold for statistical significance. Statistical analyses were performed using the IBM SPSS statistical software, version 25.0 (SPSS Inc., Chicago, IL, USA).

## RESULTS

Our cohort included 39 patients with IPAF and a mean age at ILD diagnosis of 63.2 (± 11) years. The patient’s characteristics at baseline are reported in **Table 1** and 15.4% of patients were active smokers with a mean value (±SD) of pack-years of 13 (±22.8). Almost all patients presented with dyspnoea and/or cough with a median value of MRC dyspnoea scale of 2 (±1).

**Table 1.** Patients’ baseline characteristics.

**Patients’ Characteristics**	**N = 39 (%)**
Mean age at diagnosis (±SD)	63.2 (± 11)
Female Gender	27 (69.2%)
Active smoker	6 (15.4%)
**Comorbidities**	
Obesity	3 (7.7%)
COPD / Asthma	3 (5.7%)
Arterial Hypertension	18 (46.2%)
Diabetes Mellitus	11 (28.2%)
Dyslipidaemia	12 (30.8%)
Coronary Artery Disease	4 (10.3%)
Cerebrovascular Accident	0
Osteoporosis	12 (30.8%)
Thyroid disease	16 (41%)
Malignancy	5 (12.8%)
HCV/HBV infection	1 (2.6%)
Tuberculosis	1 (2.6%)
Gastro-oesophageal Reflux Disease	2 (5.1%)

**Figure 1.**
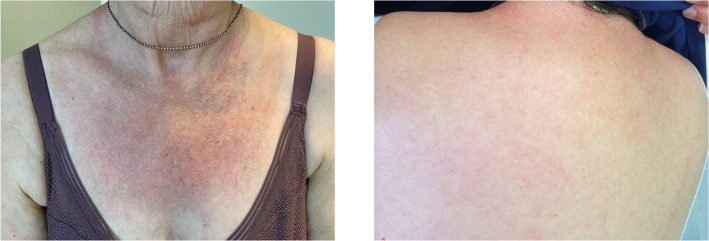
Polymorphous (a) and morbilliform (b) rash observed in two patients with IPAF included in our cohort.

Table 2 summarises patients’ features related to IPAF. Regarding the clinical domain of the IPAF criteria, the most common clinical features were arthritis (82.1%) and Raynaud’s phenomenon (25.6%). A morbilliform and/or polymorphic rash of the face, neck, and extremities (not included in the IPAF’s clinical criteria) was noted in approximately 54% of patients (**Figure 1**). ANA (59%) and anti-Ro (21%) were the most common autoantibodies. NSIP was the most prevalent radiological pattern (61.5%). Notably, 25.7% of patients in our cohort presented a radiological pattern either of UIP alone or in combination with NSIP. Treatment comprised, in all but 3 patients, corticosteroids (mean prednisolone initial dose = 24.5±18 mg) and immunosuppressants including hydroxychloroquine (23.7%), hydroxychloroquine and azathioprine (7.9%), methotrexate (23.7%), azathioprine (21.1%), mycophenolate mofetil (7.9%) and cyclophosphamide (7.9%). In our clinical practice, immunosuppressant dosage ranged from 200–400mg/d for hydroxychloroquine, 2–2.5mg/Kg/day for azathioprine, 15–25mg/week for methotrexate, 2–3g/day for mycophenolate mofetil and 6–7g total dose for cyclophosphamide (divided in monthly iv infusions).

**Table 2.** IPAF-related features.

**Clinical Domain**	**N = 39 (%)**
**Included in IPAF’s clinical criteria**	
Mechanic’s hands	5 (12.8%)
Digital ulcers	1 (2.6%)
Arthritis or polyarticular morning stiffness ≥60min.	32 (82.1%)
Palmar telangiectasias	1 (2.6%)
Raynaud’s phenomenon	10 (25.6%)
Digital oedema	1 (2.6%)
Gottron’s sign	4 (10.3%)
**Not included in IPAF’s clinical criteria**	
Sicca symptoms	14 (35.9%)
Hair loss	8 (20.5%)
Rash	21 (53.8%)
Photosensitivity	5 (12.8%)
Fever	7 (18.4%)
Mouth ulcers	1 (2.6%)
**Serologic Domain**	
ACPA	2 (5.1%)
RF (≥ 2 x upper limit of normal)	3 (7.7%)
ANA >1/320	23 (59%)
Anti-Ro/SSA	8 (20.5%)
Anti-La/SSB	2 (5.1%)
Anti-Sm	0
Anti-RNP	1 (2.6%)
Anti-Jo1	3 (7.7%)
Other autoantibodies	6 (15.4%)
**Radiological pattern – Morphologic Domain**	
NSIP	24 (61.5%)
OP	2 (5.1%)
NSIP-OP overlap	2 (5.1%)
LIP	1 (2.6%)
UIP	7 (18%)
NSIP and UIP	3 (7.7%)

### Pulmonary function tests

**Figure 2.**
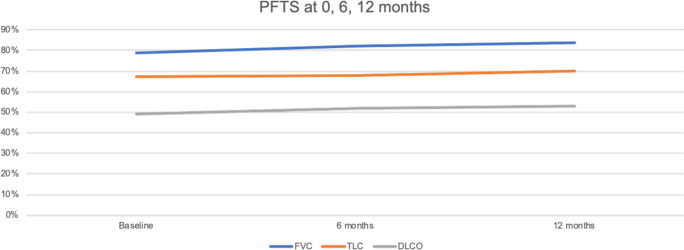
PFTs at baseline, 6 and 12 month of follow-up period. PFTs: Pulmonary Function Tests, FVC: Forced vital Capacity, TLC: Total Lung Capacity, DLCO: Diffusing capacity for carbon monoxide.

Based on PFT baseline values, our cohort consists of patients with moderate-severe impairment of pulmonary function (mean DLCO = 48.7%). In fact, PFTs performed at baseline showed a mean value (±SD) of DLCO of 48.7% (±15.9%), TLC of 67% (±11.47%) and FVC of 79% (±18.8%). Following treatment at 6 and 12 months from baseline, mean values (±SD) for DLCO, TLC and FVC were 52% (±17.16%), 67.9% (±13.6%), 81.6% (±17.6%) and 53% (±17.3%), 69.5% (±13.9%), 83.9% (±17.2%) respectively (p> 0.05) (Figure 2).

### Predictors of clinically significant deterioration

At 1 year from baseline, 20.5% of patients showed a clinically significant deterioration while 25% had a clinically significant improvement, and 54.5% showed no significant change in PFTs. A univariate analysis was performed in order to identify predictors of clinically significant deterioration taking into account baseline PFTs, Radiological pattern (UIP vs non UIP), gender, comorbidities and immunosuppressants but yielded no statistically significant results (data not shown).

### Infections

The incidence of infectious events and hospitalizations was calculated during both the first and second semester of the follow-up period. All documented infections were respiratory tract infections. During the first semester, 9 patients (23.1%) presented an infectious event and 2 (5.1%) required hospitalization. Additionally, during the second semester, 5 infections (12.8%) were reported but none resulted in hospital admission.

A univariate analysis was performed aiming at the identification of probable predictors of infectious complications in our cohort (Table 3). All tested parameters showed no statistically significant correlation with infections except for the radiological pattern. In fact, we classified the patients enrolled in our cohort based on whether they had a UIP pattern (either alone or in combination with NSIP) or a non-UIP pattern. All infections were observed in patients with a non-UIP radiological pattern (p=0.02). Further analysis in these subgroups of patients showed a trend for higher mean initial prednisolone dose = 27 (±18) mg/d in patients with non-UIP pattern versus 17 (±16) mg/d in patients with UIP pattern (p=0.4), which might explain the increased risk for infections demonstrated in patients with non-UIP pattern. Notably, no association resulted between infections and use of specific immunosuppressants.

**Table 3.** Risk factors associated with infectious complications in our cohort.

	**Infection**		**p-value**
	**(+) N=11**	**(−) N=28**	
Male gender, n (%)	2 (18.2%)	10 (35.7%)	0.28
Age >65 years, n (%)	6 (54.5%)	13 (46.4%)	0.65
BMI>30, n (%)	1 (9.1%)	2 (7.1%)	0.84
Diabetes Mellitus, n (%)	0 (0%)	11 (39.3%)	0.01
Coronary Artery Disease, n (%)	1 (9.1%)	3 (10.7%)	0.88
Malignancy, n (%)	1(9.1%)	4 (14.3%)	0.66
Radiologic pattern, n (%)			
UIP	0 (%)	10 (35.7%)	**0.02**
Non-UIP	11 (100%)	18 (64.3%)	
LTOT, n (%)	1 (9.1%)	1 (3.7%)	0.5
Initial Prednisolone dose (mg/d), mean (SD)	20 (±16.4)	26.3 (±18.5)	0.3
Baseline DLCO, mean (SD)	54.2% (±18.1)	46.7% (±14.9)	0.2
Baseline FVC, mean (SD)	72.6% (±15.83)	81.4% (±19.5)	0.18

## DISCUSSION

Herein, we report the demographic, clinical and laboratory characteristics of patients with IPAF seen in a single rheumatology centre. Of note, arthritis was the most common clinical manifestation among the clinical features of the IPAF criteria, but also, a polymorphous and or morbilliform rash – not included in the IPAF criteria – was noted in more than half of our patients. This study also offers interesting prospective data regarding the patients’ response to treatment showing stable or improved PFTs in almost 80% of patients during the first year of follow-up. Approximately 1 out of 4 patients during the first semester and 1 out of 8 patients during the second semester of follow-up had an infectious complication; two patients required hospital admission for severe respiratory tract infection during the first semester of follow-up.

The demographic-epidemiological characteristics of IPAF patients in published large retrospective series are rather heterogeneous. In fact, age at diagnosis in our cohort is similar to all other main studies^9–11^ with the exception of the study by Chartand et al.,^12^ where the reported mean age of the cohort is 54 years. Moreover, while female gender is more prevalent in both our cohort and the one described by Chartrand et al., this is not the case for most other studies.^9–11^ Similarly, active smokers represented 15.4% of our cohort, which is in keeping with the study by Chartand et al., but significantly different from the studies of Ito et al. and Oldham et al., where at least half of the enrolled patients were active or ex-smokers. Such variability reflects the retrospective nature of most published studies and further highlights the need for large prospective studies.

As to the clinical domain of the IPAF criteria, arthritis and/or morning stiffness for more than 60 minutes was the most common manifestation in our cohort with a prevalence of 82% followed by Raynaud’s phenomenon documented in 25.6% of our patients. The prevalence of arthritis is significantly higher in our cohort compared to other studies, which might be attributed to the assessment of all enrolled patients by trained rheumatologists, further highlighting the importance of multidisciplinary approach to these patients. However, Raynaud’s phenomenon is reported in similar percentages in almost all studies. Interestingly, amongst clinical features not included in the clinical domain of IPAF criteria, a morbilliform and/or polymorphic rash of the face, neck and extremities was noted in 54% of patients in our study (**Figure 1**). Albeit not specific for IPAF, the replication of this finding in other prospective cohorts might support its future inclusion in the IPAF clinical criteria. Of interest, 35.9% of the patients reported oral and/or eye dryness, which are also reported with similar prevalence in a recent prospective Italian study.^14^

Regarding the serological domain, ANA and anti-Ro/SSA autoantibodies were the most prevalent in 59% and 20.5% respectively of the patients in our study, which is in accordance with the published literature. Taken together, the high prevalence of sicca symptoms and anti-Ro/SSA antibodies suggests that secondary or associated Sjögren syndrome might be as common in IPAF as in patients with other specific CTD (eg, RA).^18^

NSIP was the most common radiological pattern, in keeping with most published studies with the exception of the cohort by Oldham et al., where most patients presented a UIP radiological pattern.^9^

Lung function was moderately to severely impaired in our patients at baseline with mean values (±SD) of DLCO, TLC and FVC at 48.7% (±15.9), 67% (±11.47) and 79% (±18.8) respectively. All but 3 of the 39 patients enrolled in this study received combined treatment with oral steroids and immunosuppressants, which resulted in stabilization or improvement of the PFTs in the majority of patients. Whilst 20.5% patients experienced a clinically significant deterioration of the PFTs our analysis failed to identify specific risk factors for such event probably due to the small sample size of our cohort. Of note, a recent Italian prospective cohort reported a clinically significant deterioration of lung function in a substantially higher percentage of their patients (34.4%) as well as significantly less patients achieving a clinically significant improvement in PFTs (6.3% vs 25% at the end of 12-month period of follow-up).^14^ Different treatment approaches among other factors might explain such discrepancies. In fact, our rationale for the management of patients with IPAF (and generally with CTD-ILD) consists on initially using medium doses of oral steroids up to 0.5mg/kg/d, aiming at rapidly reversing acute lung inflammatory lesions before evolving into fibrosis, while allowing the time for immunosuppressants to build up and eventually halt disease progression. Notably, the tapering phase of steroids starts after 2 weeks, aiming at a dose of 7.5–10mg/d of equivalent prednisolone dose at 3 months and at 0–5 mg/d at 6 months from treatment initiation. Prospective controlled or at least pragmatic studies are necessary in order to better define the most appropriate treatment strategy in these patients.

To our knowledge, this is the first study reporting the incidence of infectious complications in a cohort of patients with IPAF. Interestingly, 1 out of 4 patients in the first semester and approximately 1 out of 8 patients in the second semester had an infection, 2 of which required hospital admission. The higher incidence of infections in the first compared to the second semester might be attributed to higher steroid dose usage at the early phases of treatment as described above. Moreover, our analysis showed that all infections paradoxically occurred in patients with non-UIP pattern (p=0.02) which might be attributed to higher doses of corticosteroids used in these patients (mean initial prednisolone dose = 27 (±18) mg/d in patients with non-UIP pattern versus 17 (±16) mg/d in patients with UIP pattern, p=0.4). The rationale for using higher steroid doses in patients with non-UIP pattern is driven by the higher probability of reversing these lung lesions comparing to patients with UIP pattern.

Our study has certain limitations. Our cohort, derived by a single-centre experience, comprises a relatively limited number of patients of Greek ethnicity with short follow-up period that prevents the generalisability and reduces the power of our results. On the other hand, this study offers a detailed epidemiological and clinical description of IPAF patients, which can increase the awareness and facilitate the diagnosis of patients with IPAF. Moreover, the longitudinal prospective follow-up of these patients offers unique insights in the natural history of the disease, the response to treatment, as well as for the first time the infection rate observed in these patients.

In conclusion, this study offers a detailed description of patients with IPAF showing a high prevalence of morbilliform and/or polymorphous rash which may be considered for inclusion into IPAF criteria if further replicated in other studies. A trend of improvement in PFTs and a significant risk of respiratory tract infections mainly in the first semester of treatment and in patients with non-UIP radiological pattern were observed. Larger, prospective studies are warranted to further elucidate IPAF’s prognosis and to identify effective management approaches.
